# Reduction of artifacts from contrast media in spectral-detector CT by combined use of virtual monoenergetic images and orthopedic metal-artifact-reduction algorithm

**DOI:** 10.1097/MD.0000000000042370

**Published:** 2025-05-16

**Authors:** Nannan Pan, Junpeng Liu, Shuo Wang, Zhenwei Miao

**Affiliations:** a Department of Radiology, Tianjin Medical University Baodi Hospital, Tianjin, China; b Department of Orthopedic Surgery, Tianjin Medical University Baodi Hospital, Tianjin, China.

**Keywords:** artifacts, contrast media, orthopedic metal-artifact-reduction algorithm, spectral-detector CT, virtual monoenergetic images

## Abstract

This study assesses the artifacts reduction caused by contrast media (CM) in the subclavian and axillary veins in dual-layer spectral-detector CT using virtual monoenergetic images (VMI) and orthopedic metal-artifact-reduction (O-MAR) algorithm. A total of 61 nonconsecutive patients who underwent enhanced chest CT examinations were enrolled in the present study. Conventional images (CI), VMI, CI with O-MAR algorithm (CI + O-MAR), and VMI with O-MAR algorithm (VMI + O-MAR) were reconstructed using the same arterial CT dataset. The VMI and VMI + O-MAR images were reconstructed at 130 keV. Subjective image quality assessment was performed in terms of degree of artifacts and overall image quality using the Likert-scale. The differences in image noise, artifact index (AI) and CT number difference were compared among the 4 reconstructed images. Soft tissue adjacent to CM exhibited fewer artifacts and better image quality in VMI + O-MAR images than in VMI, CI + O-MAR images, and CI (*P* < .001). In addition, image noise and AI in VMI + O-MAR images were lower than those in VMI and CI + O-MAR images (*P* < .001). CT number difference was lower in VMI and VMI + O-MAR images than in CI + O-MAR images (*P* < .01). The CI had the highest values in image noise, AI and CT number difference (*P* < .001). The combination of O-MAR and 130 keV VMI showed a significant reduction of artifacts from CM than each technique alone and provided better image quality and diagnostic confidence. The combined application of O-MAR and 130 keV VMI can be a better alternative to reduce artifacts from CM in spectral-detector CT.

## 1. Introduction

Enhanced chest CT is increasingly used in clinical practice for the identification of pathologies by enhancing the lesion contrast.^[1]^ The power injection is usually used to push contrast media (CM) in a single syringe when performing thoracic CT.^[2]^ However, high concentration of CM in the subclavian and axillary veins may cause severe artifacts, obscuring nearby tissues, such as lesions in the upper lobes of the lung and the axillary or mediastinal lymph nodes.^[3]^ It may seriously affect the lesions detection and evaluation in cancer patients, especially those with lung cancer, considering that it is the most common cancer and may spread to the upper lobes of the lung and axillary or mediastinal lymph nodes.^[4,5]^ The accurate detection and differential diagnosis of the masses or nodules in the upper lobes of the lung or in the soft tissue (fat, muscle, blood vessel, etc) around the subclavian and axillary veins are necessary for planning appropriate treatment strategy. Therefore, it is crucial to restrain the artifacts caused by highly concentrated CM in the subclavian and axillary veins on enhanced chest CT.

High concentration of CM is severely attenuating in CT images like metal. Highly attenuating materials can cause 3 different kinds of artifacts: (1) Beam hardening: a particular problem of high physical density and atomic number materials. The CT X-ray beam is polychromatic, and low-energy photons are absorbed more easily than high-energy photons. (2) Photon starvation: only a few photons reach the detector. (3) Scattering: direction changes of the photos and ending up in different detectors.^[6–9]^ Virtual monoenergetic images (VMI) reconstruction is a powerful method to reduce the beam hardening artifacts.^[10]^ The dual-layer spectral-detector CT can split polychromatic CT X-ray beam into high- and low-energy photos, which are then absorbed by 2 different layers of detectors, respectively.^[10,11]^ High- and low-energetic images can be linearly combined to get the VMI, which are similar to conventional polyenergetic images.^[10,12]^ High keV images have been investigated as a solution to reduce beam-hardening artifacts.^[13–15]^

Recently, 2 studies have reported optimal keV images to reduce the artifacts caused by high concentration of CM. Laukamp et al^[16]^ explored the role of VMI in the reduction of artifacts from CM in axillary and subclavian veins, and recommended a keV range of 100 to 160 keV and an appropriate energy level of 130 keV for the best diagnostic assessment. Kim et al^[17]^ reported that the optimal energies of VMI for decreasing artifacts due to CM were 100 and 130 keV. Both studies demonstrated that the high-energy level of virtual monochromatic images could reduce beam-hardening artifacts from CM. Orthopedic metal-artifact-reduction (O-MAR) algorithm adopts an iterative loop to subtract the output correction image from the original input image and the resultant image can then become the new input image.^[18]^ The O-MAR can reduce artifacts caused by highly attenuating materials in CT images.^[18]^ Previous studies reporting on metal artifacts reduction for orthopedic implants found that O-MAR or the combination of VMI and O-MAR could reduce artifacts and improve image quality.^[19,20]^ However, only 1 study verified whether the combination of VMI and O-MAR could further reduce artifacts from CM.^[21]^ Therefore, in this study, we aimed to evaluate the effect of VMI, O-MAR algorithm and their combination on the reduction of artifacts from CM by using objective and subjective assessments and hypothesized that images reconstructed with VMI and O-MAR together may further reduce artifacts and improve image quality.

## 2. Methods

### 2.1. Patient population

The local institutional review board approved this retrospective study, and informed consent was waived. The inclusion criteria were as follows: (1) contrast-enhanced thoracic staging or restaging examinations from July 2019 to January 2022; (2) artifacts caused by highly concentrated CM impairing the diagnostic value of conventional CT images; and (3) age ≥ 18 years. According to the criteria, a total of 61 nonconsecutive patients were included in this study. The criteria were applied by a radiologist with 6 years of experience in chest imaging diagnosis.

### 2.2. CT protocol

All patients were examined using the dual-layer spectral-detector CT (IQon Spectral CT, Philips Healthcare). The images were obtained from the thoracic inlet level to the lung base during a single aspiratory breath-hold with the following parameters: tube voltage, 120 kVp; scan options, helix; collimation, 64 × 0.625 mm; slice thickness, 0.67 mm; slice increment, 0.5 mm; pitch, 0.98; matrix = 512 × 512; rotation time, 0.5 second; and tube current modulation, 3D modulation. Iodinated CM (Ioversol Injection, 350 mgI/mL) was injected into the right (n = 45) or left (n = 16) antecubital vein using a power injector (Stellant, Medrad) in all patients with the injection rate of 3 mL/s.

Conventional images (CI) were reconstructed using hybrid iterative reconstruction (iDose^4^, level 3; Philips Healthcare).^[22]^ Based on previous studies, the reconstructed energy level was set at 130 keV.^[16,17]^ VMI at 130 keV were reconstructed using the spectral iterative reconstruction algorithm (Spectral B, level 3; Philips Healthcare). CI + O-MAR images were reconstructed using an iterative metal-artifact-reduction algorithm (O-MAR, Philips Healthcare) based on CI. VMI reconstructed with O-MAR were referred to as VMI + O-MAR.

### 2.3. Objective image analysis

**Figure 1.**
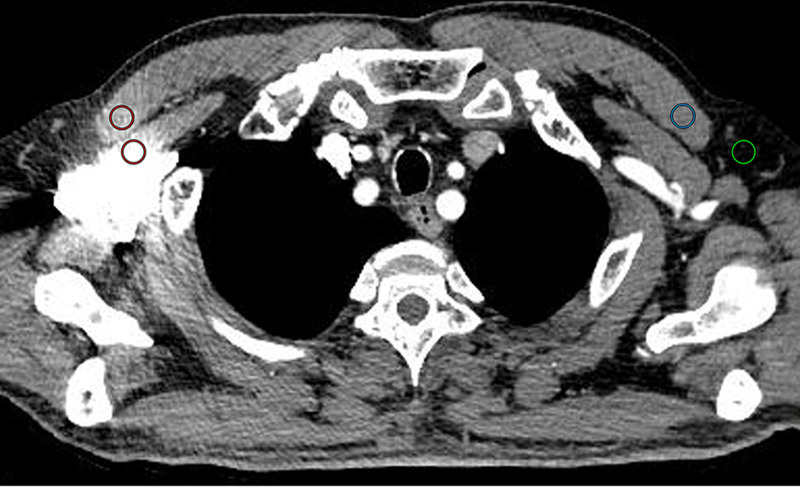
Location of ROIs for the objective image analysis. ROIs were drawn in pectoralis major muscle and pectoralis minor muscle close to the CM and most affected by artifacts (red ROIs). ROI was drawn in pectoralis major muscle on the contralateral side which it was not affected by artifacts (blue ROI). Furthermore, ROI was drawn in the artifact-free subcutaneous fat layer (green ROI). ROIs (40–60 mm²) were placed on CI and copied to VMI, CI + O-MAR and VMI + O-MAR images to guarantee the same size, shape and slice. CI = conventional images, CM = contrast media, O-MAR = orthopedic metal-artifact-reduction, ROIs = regions of interest, VMI = virtual monoenergetic images.

Objective image analysis was performed by drawing regions of interest (ROIs) on axial arterial CT images (slice thickness, 1 mm) in the soft tissue window (window width, 360; window level, 60). ROIs were drawn in the pectoralis major and pectoralis minor muscles adjacent to the subclavian and axillary veins and most affected by artifacts (named artifact areas), the pectoralis major muscle on the contralateral side where it was not affected by artifacts (named artifact-free area), and the artifact-free subcutaneous fat layer. The size of 4 ROIs was set to 40 to 60 mm² (Fig. 1) and could be adjusted depending on the muscle size. ROIs were first drawn in CI images and then copied onto VMI, CI + O-MAR and VMI + O-MAR images to maintain the size, shape and slice. The CT number (HU) and standard deviation (SD) value (HU) within the ROIs were recorded and averaged. The SD value of the artifact-free subcutaneous fat layer was calculated to quantify image noise. To assess the degree of artifacts, the artifact index (AI) was calculated with the following formula: $AI=\sqrt{SD1^{2}-SD2^{2}}$, where SD1 value and SD2 value represented SD value of the artifact area and the artifact-free area, respectively. CT number difference between pectoralis major muscle adjacent to the subclavian and axillary veins and the contralateral side was compared.

### 2.4. Subjective image analysis

For the subjective image analysis, all arterial CT images were evaluated by 2 radiologists with 6 years of experience in thoracic radiology under the following parameters: slice thickness of 1 mm; axial plane; window level of 60; window width of 360; observers were encouraged to adopt appropriate window settings. Each reviewer rated the artifacts on a 5-point Likert scale: 1, imperceptible artifacts; 2, minor artifacts; 3, moderate artifacts; 4, pronounced artifacts; and 5, severe artifacts.^[23]^ The overall image quality including the upper lobes of the lung, axilla and mediastinum around the subclavian and axillary veins was assessed using the Likert ranking described as 1 to 5 grades (1 = completely diagnostic, 5 = severely impaired reliability). Previous studies have reported that O-MAR might reveal new and unexpected artifacts, resulting in image distortion.^[20,24]^ In this study, the degree of new artifacts was assessed as follows: 1, no new artifacts and image distortion; 2, minor; 3, moderate; 4, pronounced; and 5, severe.

### 2.5. Statistical analysis

Because of the non-normal distribution of the data, the nonparametric approach, Friedman rank-sum test, was performed to compare the differences in subjective image scores, image noise, AI and CT number difference among the 4 reconstructed images. Pairwise comparisons were performed using the Wilcoxon signed-rank test. Furthermore, for subjective analysis, the intraclass correlation coefficient was performed and interpreted as described previously.^[25]^ IBM SPSS Statistics for Windows, Version 23.0 (Armonk, NY: IBM Corp.) was used for statistical analyses. Statistical significance was set at *P* < .05.

## 3. Results

### 3.1. Patients

In this study, 61 patients were included (39 men and 22 women; mean age, 64.4 ± 10.3 years; range, 40–89 years). There were 40 patients with lung cancer, 9 patients with colorectal cancer, 3 patients with malignant thymoma, 2 patients with esophageal cancer, 1 patient with stomach cancer, 2 patients with cervical cancer and endometrial cancer, 1 patient with breast cancer, 1 patient with pancreatic cancer, 1 patient with lung cancer and colon cancer, and 1 patient with metastatic tumors.

### 3.2. Objective image quality

**Figure 2.**
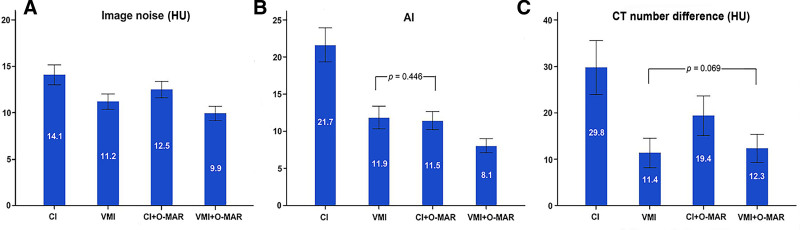
Bar charts showing the image noise (A), AI (B), and CT number difference (C) in the 4 reconstructed images (CI, VMI, CI + O-MAR, and VMI + O-MAR). For the image noise, all pairs showed significant differences (*P* < .001). There were significant differences in AI among the 4 reconstructed images (*P* < .001), except between VMI and CI + O-MAR image (*P* < .446). There were significant differences in CT number difference (*P* ≤ .006), except between VMI and VMI + O-MAR images (*P* = .069). AI = artifact index, CI = conventional images, O-MAR = orthopedic metal-artifact-reduction, VMI = virtual monoenergetic images.

The differences in image noise, AI and CT number difference among the CI, CI + MAR, VMI, and VMI + MAR images were statistically significant (*P* < .001) (Fig. 2). Image noise of CI, VMI, CI + O-MAR, and VMI + O-MAR images were 14.1 ± 4.2, 11.2 ± 3.2, 12.5 ± 3.4, and 9.9 ± 3.0 HU, respectively. AI of CI, VMI, CI + O-MAR, and VMI + O-MAR images were 21.7 ± 9.0, 11.9 ± 6.0, 11.5 ± 4.8, and 8.1 ± 3.8, respectively. The CT number difference of CI, VMI, CI + O-MAR, and VMI + O-MAR images were 29.8 ± 22.7, 11.4 ± 12.4, 19.4 ± 16.7, and 12.3 ± 11.8 HU, respectively. Post hoc test showed significant differences in image noise, AI and CT number difference between CI and the other 3 reconstructed images (all *P* < .001). The noise and AI of VMI + O-MAR images were lower than that of CI + O-MAR and VMI (all *P* < .001). The CT number difference of VMI and VMI + O-MAR images were lower than that of CI + O-MAR (VMI vs CI + O-MAR: *P* < .001; VMI + O-MAR vs CI + O-MAR: *P* < .01), and there was no statistical difference between the VMI and VMI + O-MAR images (*P* = .069).

### 3.3. Subjective image quality

**Figure 3.**
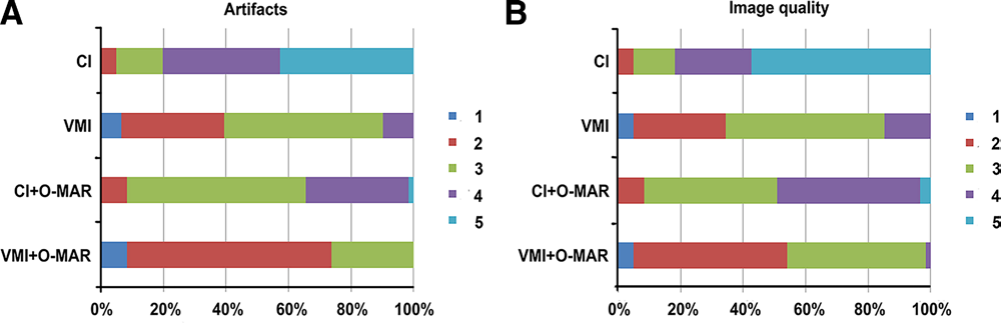
Subjective image quality scores of the 4 reconstructed images (CI, VMI, CI + O-MAR, and VMI + O-MAR). VMI + O-MAR showed the lowest degree of artifacts (A) and the best overall image quality (B), and had more images that were graded 1 and 2. The scores of VMI, CI + O-MAR, and VMI + O-MAR were significantly lower than CI. CI = conventional images, O-MAR = orthopedic metal-artifact-reduction, VMI = virtual monoenergetic images.

**Figure 4.**
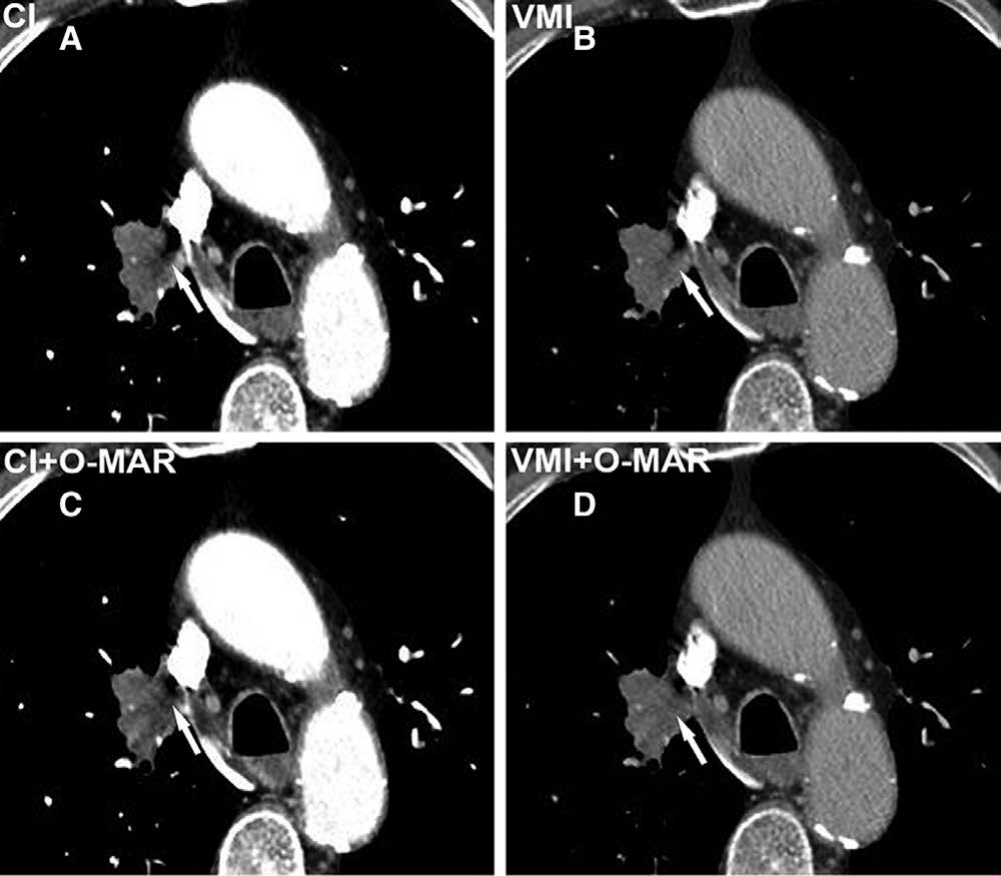
A 74-year-old man with lung cancer. Enhanced chest CT images show an irregular mass in the right lung near the mediastinum. On CI (A), it is hard to evaluate the intralesional density and the relationship with mediastinum due to the artifacts from CM in the superior vena cava (arrows). The VMI (B) and CI + O-MAR (C) reduce some artifacts and improve the image quality. The VMI + O-MAR image (D) shows a mass with homogenous density that has not invaded the mediastinum. Subjective assessments from both readers generated the best scores for the VMI + O-MAR image. CI = conventional images, CM = contrast media, O-MAR = orthopedic metal-artifact-reduction, VMI = virtual monoenergetic images.

**Figure 5.**
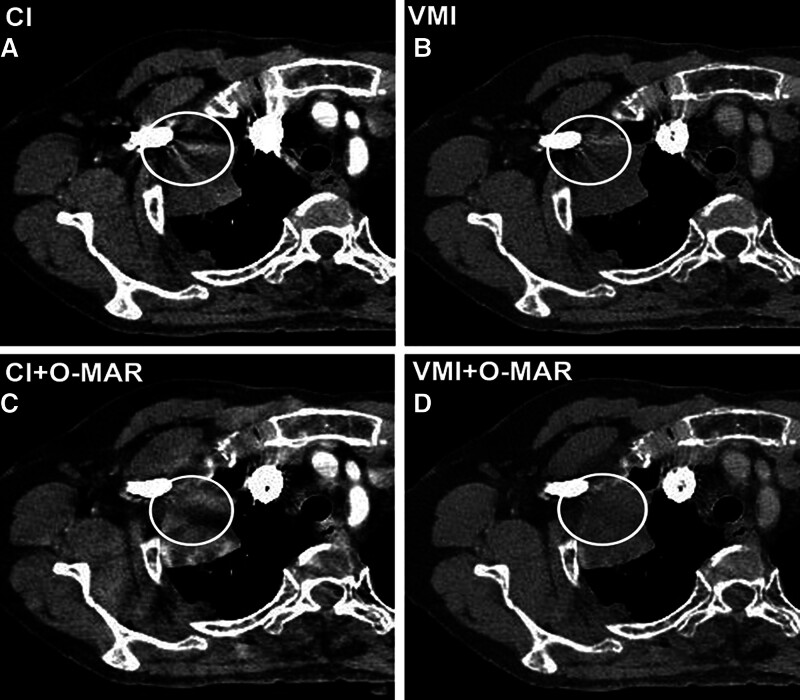
An 83-year-old woman with a soft tissue mass in the right upper lobe. Enhanced chest CT images show a soft tissue mass with irregular margin and homogeneous density (circles). On CI (A), it is difficult to assess the mass because of the severe artifacts due to CM in the subclavian vein. VMI (B) reduces image artifacts to some extent, but some remaining artifacts are still visible. The CI + O-MAR image (C) reduces the artifacts, but some new streak artifacts are observed. However, the VMI + O-MAR image (D) reduces most of the artifacts and enables the evaluation of the shape, margin, and density of the mass. CI = conventional images, CM = contrast media, O-MAR = orthopedic metal-artifact-reduction, VMI = virtual monoenergetic images.

**Figure 6.**
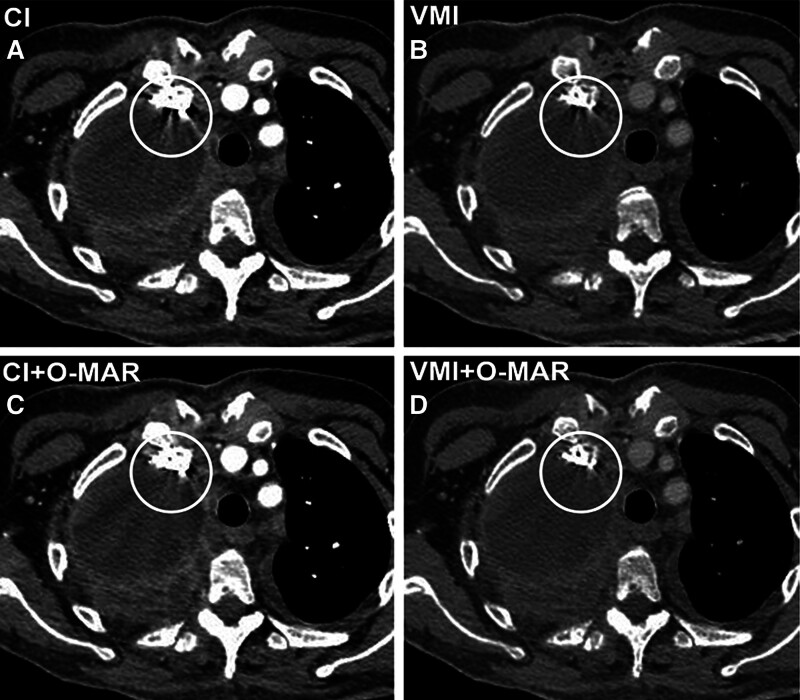
A 65-year-old woman with lung cancer. Enhanced chest CT images show pleural effusion and pleural thickening (circles). Artifacts caused by CM in the superior vena cava are the most severe on CI (A). VMI (B), and CI + O-MAR image (C) reduced some artifacts. VMI + O-MAR image (D) obtains the best subjective assessment scores from both reviewers. CI = conventional images, O-MAR = orthopedic metal-artifact-reduction, VMI = virtual monoenergetic images.

**Figure 7.**
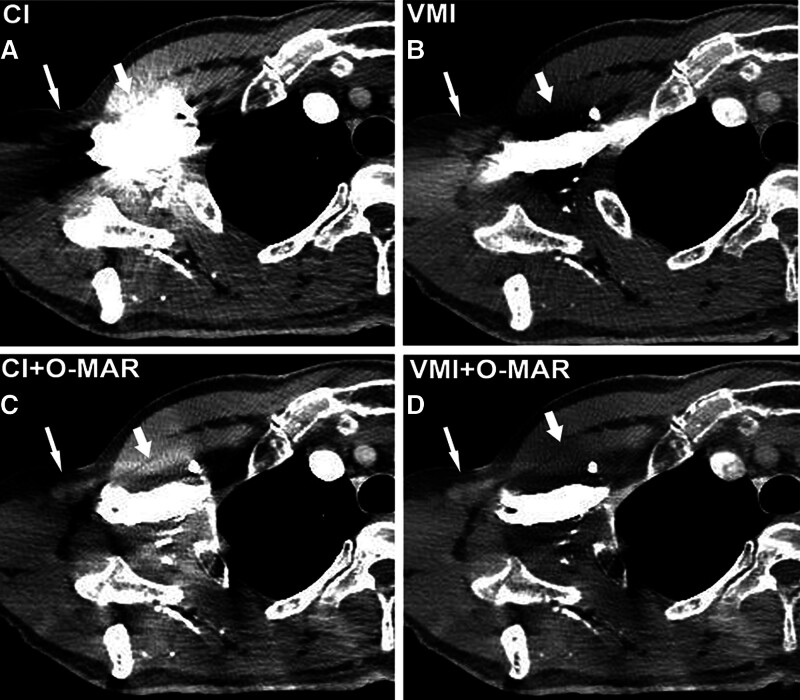
A 54-year-old man with an axillary lymph node. On CI (A), the axillary lymph node (long arrow) and adjacent soft tissue (short arrow) are difficult to evaluate because of the artifacts caused by CM in the subclavian vein. The VMI (B) and CI + O-MAR image (C) reduce some artifacts, but photo starvation artifacts and new streak artifacts are observed on VMI and CI + O-MAR image, respectively. The artifacts around the soft tissue are further reduced and the lymph node is clearly depicted in the VMI + O-MAR image (D). CI = conventional images, CM = contrast media, O-MAR = orthopedic metal-artifact-reduction, VMI = virtual monoenergetic images.

In this study, subjective assessment supported the results of the objective image quality assessment. Compared to CI, a significant reduction of artifacts was found in CI + O-MAR images and VMI (both *P* < .001), and VMI + O-MAR showed the most reduction of artifacts (all *P* < .001). VMI + O-MAR images had the best overall image quality (all *P* < .001), and VMI, CI + O-MAR were both better than CI (both *P* < .001) on a 5-point Likert scale (Table 1 and Fig. 3). In the subjective assessment, new steak artifacts produced by O-MAR that may cause image distortions were noticed (Table 1). However, the new streak artifacts led to mild impairment on the image quality according to the 5-point Likert scale. The intraclass correlation coefficients between the 2 radiologists for degree of artifacts, overall image quality and new streak artifacts were 0.989, 0.980, and 0.993 respectively. Figures 4–7 showed the subjective assessment of CI, VMI, CI + O-MAR, and VMI + O-MAR images.

**Table 1:** Subjective assessment of artifacts and image quality.

	Artifacts reduction	Image quality	New artifacts
CI	4 (3–5)	5 (3–5)	
VMI	3 (2–4)	3 (2–4)	
CI + O-MAR	3 (3–4)	3 (3–4)	2 (1–3)
VMI + O-MAR	2 (2–3)	2 (2–3)	1 (1–2)

CI = conventional images, O-MAR = orthopedic metal-artifact-reduction, VMI = virtual monoenergetic images.

## 4. Discussion

In this study, we applied VMI, O-MAR algorithm and their combination to reduce artifacts from CM in subclavian and axillary veins in enhanced chest CT imaging. Objective results revealed that all 3 techniques reduced image noise, AI and CT number difference. Furthermore, the combination of VMI and O-MAR minimized image noise and AI. VMI exhibited the lowest CT number difference, and there was no statistical difference between the VMI and VMI + O-MAR. Subjective assessment supported objective results showing that all 3 techniques reduced artifacts and improved image quality. The application of VMI and O-MAR yielded the best results.

We investigated patients with different types of neoplasms receiving dedicated staging and restaging examinations. Artifacts reduction of CM and appropriate assessment of the tumors or nodules located in the upper lobes or the surrounding soft tissue can be fairly important in the particular patient population. Our study demonstrated that subjective artifacts reduction scores and overall image quality scores reduced after applying either VMI or O-MAR compared to CI, and further reduced after using VMI and O-MAR in combination. These results were consistent with previous studies focusing on reducing metal artifacts, which indicated that the combination of VMI and O-MAR could provide better subjective image quality and artifacts reduction than when they were used alone.^[20,26]^

VMI at 40 keV generates the highest CT number, because the energy level is closest to the K-edge of iodine (33.2 keV).^[27]^ VMI of high keV values can increase the distance to K-edge of iodine and reduce CT number,^[28,29]^ therefore, they should be helpful in reducing artifacts from CM. Currently, the well-established method is to use high-energy VMIs to reduce metal artifacts of implants or prostheses in different areas of the body, mainly in dentistry and orthopedics.^[30–33]^ Recently, 2 studies applied VMI from spectral CT to reduce artifacts caused by CM in enhanced chest CT and explored the appropriate range of keV values to maximize image quality.^[16,17]^ Spectral-detector CT can balance the CT number and image noise, but as the keV value increases, iodine-soft tissue contrast decreases. Finally, VMI with a relatively high keV value, 130 keV, was used in our study to assess the artifacts reduction and image quality in enhanced chest CT examinations and revealed better results than CI, regardless of the objective or subjective analysis.

To maintain soft tissue contrast, the O-MAR algorithm was used in our study. Previously, 2 studies for reducing metal artifacts in patients with hip prostheses showed that O-MAR provided higher soft tissue contrast than VMI.^[19,34]^ O-MAR, as an independent method, reduced image artifacts from CM compared to CI, but it was less effective than applying VMI alone. O-MAR algorithm was almost used to reduce metal artifacts before. For example, O-MAR algorithm can correct hypodense artifacts caused by beam hardening in hip and knee arthroplasty.^[19,35]^ Our study results revealed that O-MAR has the potential to reduce artifacts of CM with high physical density and atomic number. However, there were new artifacts and no significant improvement in subjective assessment. Based on the operating principle described in the introduction section, O-MAR algorithm can create images that do not include pixels categorized as high atomic number materials, such as mental or CM.^[18]^ The loss of projection data of high atomic number materials may decrease the spatial resolution and further result in new streak artifacts.^[9]^ Previous studies have also reported the new streak artifacts produced by O-MAR.^[19,36]^ Almost all the patients showed new streak artifacts related to the O-MAR algorithm in our study, however, the low scores of the new artifacts suggested that these artifacts did not cause obvious image impairment.

Finally, we combined O-MAR with VMI, to reduce CM artifacts and obtained satisfactory results. Applying VMI and O-MAR together provided better objective and subjective image qualities than each algorithm alone. To the best of our knowledge, only 1 study has applied O-MAR, VMI and their combination to reduce artifacts from CM and revealed similar results to our study. They demonstrated that VMI alone and its combination with O-MAR offered appropriate approaches for artifact reduction.^[21]^ In accordance with this previous study, we applied image noise and CT number difference to assess image quality objectively. Furthermore, we supplemented a different objective evaluation parameter, AI, to assess the degree of artifacts. AI is effective and widely used to assess artifacts.^[20,37]^ Our results supported and supplemented their conclusions. Therefore, we recommended combining VMI and O-MAR as a supplement to CI to improve image quality, especially in conditions with severe artifacts from CM.

In the future, there will be numerous possibilities to reduce CM artifacts with the evolution of CT technology. For example, the clinical photon-counting CT has advantages of reduced doses of CM, increased iodine signal and improved spatial resolution,^[38]^ which may help reduce CM artifacts in axillary and subclavian veins. Moreover, optimization of injection protocols such as injecting CM followed by a saline flush, could be an efficient approach to reduce artifacts from CM.^[39,40]^

This study has several limitations. First, we selected only 130 keV in this study. Previous studies have already verified that high keV can reduce beam hardening artifacts from CM, and the optional keV value range for image quality was 100 to 160.^[16,17,21]^ By further increasing the keV levels, an overcorrection of artifacts and a decrease of tissue contrast may occur.^[16]^ Therefore, only 130 keV was chosen for this study. Second, iterative reconstruction algorithms themselves affect the objective evaluation to some extent. To improve the assessment of image quality, detailed subjective evaluations by 2 senior radiologists were performed in this study. Finally, the number of samples recruited in this study was relatively small. Further studies with larger populations should be conducted to confirm our findings.

## 5. Conclusions

In conclusion, the combination of O-MAR and 130 keV VMI could improve image quality and reduce artifacts caused by CM in axillary and subclavian veins on enhanced chest CT compared to CI. O-MAR reduced the artifacts significantly while causing new streak artifacts. The combination of O-MAR and VMI provided better subjective and objective image quality than each technique alone. The images reconstructed by VMI and O-MAR should be used as supplements to the CI.

## Author contributions

**Conceptualization:** Nannan Pan, Zhenwei Miao.

**Data curation:** Shuo Wang.

**Formal analysis:** Nannan Pan, Junpeng Liu, Shuo Wang.

**Methodology:** Nannan Pan, Junpeng Liu, Shuo Wang.

**Writing – original draft:** Nannan Pan.

**Writing—review & editing:** Shuo Wang, Zhenwei Miao.
